# Ionic Tuning of Droplet Motion on Water Surface

**DOI:** 10.3389/fchem.2019.00788

**Published:** 2019-11-19

**Authors:** Yudai Mikuchi, Hirofumi Yamashita, Daigo Yamamoto, Erika Nawa-Okita, Akihisa Shioi

**Affiliations:** Department of Chemical Engineering and Materials Science, Doshisha University, Kyoto, Japan

**Keywords:** droplet oscillation, oil/water interface, di(2ethylhexyl)phosphate, spatiotemporal pattern, ionic tuning

## Abstract

Herein, the oscillation of an oil droplet on the surface of water is studied. The droplet contains an anionic surfactant that can react with the cations present in water. The oscillation starts after a random motion, and the oscillation pattern apparently depends on the cation species in the water phase. However, a common pattern is included. The cation species only affects the amplitude and frequency and sometimes perturbs the regular pattern owing to the instability at the oil/water interface. This common pattern is explained by a simple model that incorporates the surfactant transport from the droplet to the surrounding water surface. The dependency of the amplitude and frequency on cation species is expressed quantitatively by a single parameter, the product of the amplitude and square of frequency. This parameter depends on the cationic species and can be understood in terms of the spreading coefficient. The simple model successfully explains this dependency.

## Introduction

A droplet on a liquid surface often shows various spatiotemporal pattern formations. Recently, the number of studies focusing on droplet dynamics under a non-equilibrium state has increased, where the non-equilibrium is caused by mass transport and/or chemical reactions (Ye et al., 1994; Shioi et al., 2003; Sumino et al., 2005; Lagzi et al., 2010; Pimienta et al., 2011; Ban et al., 2013; Hermans et al., 2013; Banno and Toyota, 2015; Seemann et al., 2016; Zwicker et al., 2016; Seyboldt and Jülicher, 2018). This is one of the simplest pattern formations in non-equilibrium open systems. The studies in this field may be associated with the physicochemical understanding of the spatiotemporal patterns in living systems (Zwicker et al., 2016; Seyboldt and Jülicher, 2018) and application of non-linear dynamics to chemical processes such as wetting (Wang et al., 2015; Zhang et al., 2015), drying (Ok et al., 2016; Janssens et al., 2017), and active transport (Bottier et al., 2009; Goto et al., 2015). Furthermore, these studies potentially lead to the design of abiotic chemical where evaporation produces the non-equilibrium state (Stocker and Bush, 2003; Pimienta et al., 2011; Antoine and Pimienta, 2013). However, the chemical control of such droplet dynamics is an attractive topic because it can yield its chemo-responsive nature. Only a few studies have been reported for the chemical control of droplet dynamics on a liquid surface (Lagzi et al., 2010; Ban et al., 2013), and how the droplet dynamics under evaporation is affected by chemistry is not fully elucidated. In general, the interplay between the mass transport and chemical reactions is a key topic in the design of biomimetic systems. However, this is not fully studied for droplet dynamics on a liquid surface despite it being the simplest system.

An oil/water interface containing bis(2-ethylhexyl) phosphate (DEHPA) shows chemo-sensitive instability (Hosohama et al., 2011; Miyaoka et al., 2012). Some types of cations cause a spontaneous convection by chemical reactions with DEHPA, whereas other types of cations do not. Even among the cations causing instability, the characteristics of the convection depend on the cation species. When a float is placed at an oil/water interface, its motion can be regulated by the cation species in water (Yasui et al., 2015). Such an oil/water system is very much suitable to study the chemical control of droplet dynamics. In the present study, the dynamics of an oil droplet containing DEHPA on a water dissolving electrolyte is investigated: The oil droplet with DEHPA spontaneously moves on the water surface. It shows a regular oscillation with some types of cations, whereas it is perturbed or violated by other types of cations. The frequencies and amplitudes of the regular oscillations depend on the cation species. This dependency can be explained by a simple model, in which the cation effect is considered in terms of the effects on the surface and interfacial tension.

## Materials and Methods

DEHPA (> 97%) was purchased from Sigma-Aldrich Co. LLC. Silicone oil was purchased from Shin-Etsu Chemical Co., Ltd. Other chemicals were reagent grade and purchased from Wako Pure Chemical Industries, Ltd. CaCl_2_·2H_2_O, FeCl_2_·4H_2_O, FeCl_3_·6H_2_O, SrCl_2_·6H_2_O, MgCl_2_·6H_2_O, MnCl_2_·4H_2_O, and CoCl_2_·6H_2_O were used as electrolytes. Decane was used as the oil phase (Silicone oil was also used for a control experiment). DEHPA and each electrolyte were dissolved in the oil and water phases, respectively. The concentrations of DEHPA and electrolyte were 100 and 10 mM, respectively. Subsequently, 10 mL of water phase was poured into a petri-dish of 48 mm diameter, after which an oil droplet of 3 μL was placed on the water surface. The droplet motions were monitored by a single-lens reflex camera (Canon EOS Kiss X9) with 30 fps frame rate. Digital microscope VW-6000 (Keyence corporation) with a frame rate ranging from 30 to 250 fps was also used when a higher time resolution was required. The movie was analyzed by image analysis software Ulead Video Studio (Corel Corporation) and TEMA (Photron Limited).

We also measured the droplet diameter under equilibrium. Then, 10 mL of the water phase was poured in another petri-dish of 48 mm diameter. Just after an oil droplet of 3 μL was placed on the water, this petri-dish was covered to avoid evaporation to outside environment. The petri-dish fogged owing to the evaporation of the oil in the petri-dish. Thus, we used the shadow graph technique to measure the droplet diameter. The diameter after 300 s was measured. The single-lens reflex camera (Canon EOS Kiss X9) with 30 fps frame rate was also used for this experiment.

The surface and interfacial tensions were also measured. In these experiments, the oil and water phases, the volume of which were 10 mL, were equilibrated. After the equilibration, the surface and interfacial tensions were measured by a pendant drop technique with one attention (Biolin Scientific Holding AB). A drop of a higher density fluid was formed in a lower density fluid. Spreading coefficient *S* was calculated from these surface and interfacial tensions.

The evaporation rate of oil was also measured from the weight loss of the oil phase. Then, 5 mL oil (decane or silicone oil) was poured in a petri-dish of 48 mm diameter. The petri-dish was set on an electronic balance to measure its weight every 5 min. We also observed the convection pattern in the water phase on which an oil droplet was placed. Separately, carbon powder (~3 mg) was dispersed in 10 mL of water phase as tracer particles. This water phase was poured in a petri-dish of 48 mm diameter. A core of a mechanical pencil (Mitsubishi Pencil Co., Ltd. uni.3-202ND) was inserted vertically across the water surface. An oil droplet of 3 μL was placed on the water surface around this inserted pillar. The droplet migration was restricted so that a stable convection pattern was observed. The motion of the tracers was monitored by the single-lens reflex camera (Canon EOS Kiss X9) at a frame rate of 30 fps. Particle image velocimetry analysis was performed (LaVision, DaVis 8). All the experiments were performed at room temperature (20–25°C).

## Results

**Figure 1:**
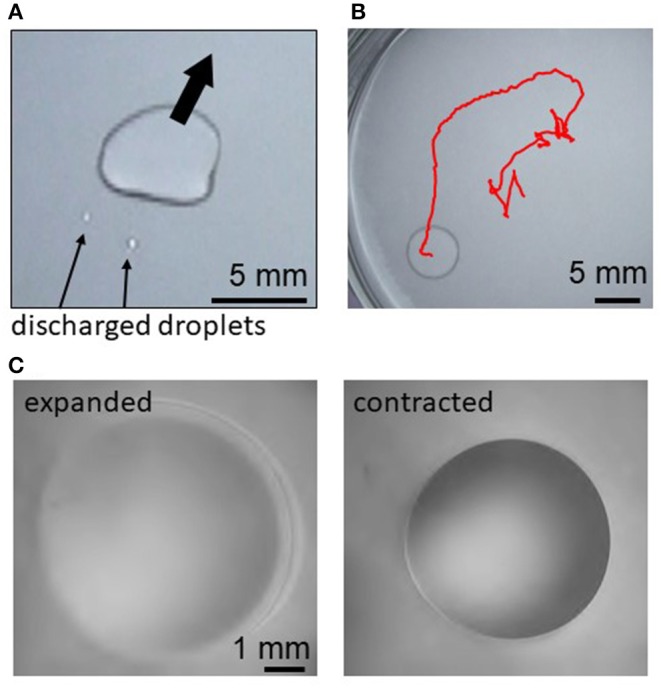
Droplet motion and oscillation for Mg^2+^, **(A)** discharge of smaller droplets after film expansion, **(B)** trajectory of the random motion for 140 s, and **(C)** expanded and contracted states during the periodic regular oscillation.

Figure 1 shows examples of droplets motions. Just after a decane droplet is placed, a thin film begins to expand from the droplet periphery. This is observed for the cations except Fe^3+^. Smaller droplets are sometimes discharged from a part of the film edge (Figure 1A). Then, the original droplet moves toward the opposite direction of this discharge. This motion is repeated, resulting in a random motion (Figure 1B) and deformation (Figure 1A) of the droplet. When Fe^2+^ or Ca^2+^ are dissolved in water, this random motion continues until the droplet disappears by transformation into a film and/or smaller droplets, evaporation, and dissolution. When Mg^2+^, Sr^2+^, Mn^2+^, or Co^2+^ is dissolved, the random motion gradually weakens. After it stops, the droplet starts to exhibit an oscillatory shape change between expanded and contracted shapes (Figure 1C). (See Supplementary Video 1 for all the cations and Supplementary Video 2 for Mg^2+^ for the regular oscillation regime.) When Fe^3+^ is dissolved in water, the oil droplet only expands on water and disappears by transforming into a film, evaporation, and dissolution.

**Figure 2:**
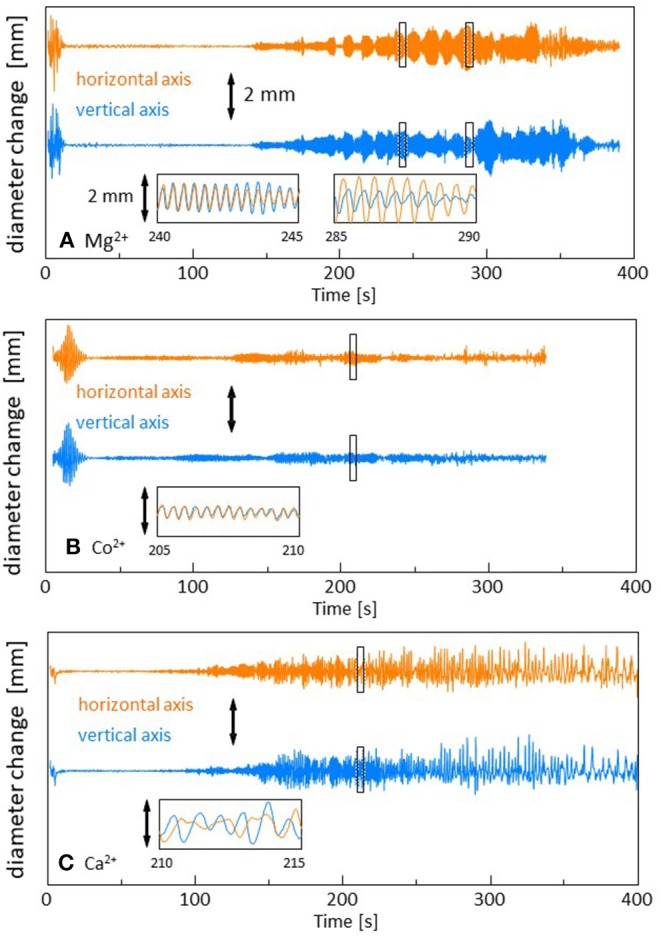
Change in the droplet diameter. The drift in the droplet size over a long time is subtracted. Three types of dynamics are shown. **(A)** Mg^2+^ with a regular oscillation, **(B)** Co^2+^ with a quasi-regular oscillation, and **(C)** Ca^2+^ with an irregular fluctuation. The insets show the expanded versions of the marked squares. The scale bar is 2 mm in all the figures.

The time course of the droplet diameter is shown in Supplementary Figure 1, in which we see weak drifts and discontinuous changes in the droplet diameter. These weak drifts and discontinuous changes are subtracted in Figure 2 because they are caused by an error in the treatment of the video files in the software. The diameters are measured along the two orthogonal directions in the video frame. For Mg^2+^ (and Sr^2+^and Mn^2+^), one can see the time range within which the droplet shape oscillates quite regularly (140 s−390 s in Figure 2A). The oscillation amplitude changes over a long time, during which the envelopes oscillate with a long period (beat). Though a similar oscillation is observed for Co^2+^ (90 s−280 s in Figure 2B), the regularity of the pattern, as shown in the inset, is slightly perturbed. Here, we refer it as a quasi-regular oscillation. For Ca^2+^ (and Fe^2+^), the diameter changes irregularly throughout the experiment (90 s−400 s in Figure 2C). This irregular diameter change is similar to that reported by Stocker and Bush (2003). A regular oscillation of a floating droplet was also reported by Antoine and Pimienta (2013). The droplet oscillations reported until now have been caused by the evaporation of the oil (Stocker and Bush, 2003; Antoine and Pimienta, 2013), and the patterns were dependent on the oil species and evaporation rate. In the present system, the different types of droplet dynamics are generated by an appropriate selection of cations (The results for the cations that are not presented in Figure 2 are shown in Supplementary Figure 2).

**Figure 3:**
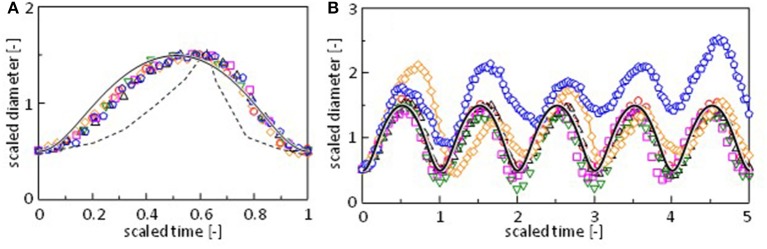
Scaling of the oscillation pattern for **(A)** a single pulse and **(B)** five pulses. The oscillation is taken around 225 s (○, Mg^2+^), 485 s (Δ, Mn^2+^), 250 s (□, Sr^2+^), 205 s (▿, Co^2+^), 214 s (♢, Ca^2+^), and 154 s (pentagon, Fe^2+^) in Figure 2 and Supplementary Figure 2. Dashed curve is reproduced from the literature (Pimienta et al., 2011). Solid curve is calculated by a model (Figure 7A).

One period of the diameter change, i.e., the period between two neighboring local minima, is scaled appropriately and superposed in Figure 3A. For Mg^2+^, Sr^2+^, and Mn^2+^, a pulse in the regular oscillation regime is selected. A pulse in the quasi-regular regime is selected for Co^2+^. For Fe^2+^ and Ca^2+^, a pulse is randomly selected from the fluctuation pattern. The result shows that the diameter change has a common pattern independent of the cation species. Figure 3B shows the scaling for five periods. Figures 3A,B indicate that even irregular shape changes in case of Ca^2+^ and Fe^2+^ exhibit a common pattern. For these cations, fluctuations in the amplitude and periodicity produce randomness. This common pattern is different from the oscillation pattern reported by Antoine and Pimienta, which is superposed in Figure 3A (Antoine and Pimienta, 2013).

The similarity in the droplet dynamics with film formation and droplet discharge reported until now is discussed in terms of hydrodynamics, in which the evaporation of oil plays a crucial role (Karapetsas et al., 2011). Here, the surface flow to maintain the film is associated with the droplet motions: The surface flow transports the surfactant molecules and modulates the interfacial (surface) tension and contact angle of a sessile lens (Antoine and Pimienta, 2013). In the present study, the measurement of the weight loss of decane reveals that the evaporation flux of decane is ~1.74 × 10^−6^ g/mm^2^, resulting in 0.3% weight loss in petri-dish during 1 h (Supplementary Figure 3). This evaporation rate appears to be very much smaller than for the dichloromethane droplet reported previously. Despite its low volatile nature, the decane droplet can exhibit the oscillatory shape change. To examine the effects of evaporation further, silicone oil was used. We confirmed that silicone oil does not evaporate during the experimental time range (Supplementary Figure 3). For all the cations used in this study, outstanding motion and shape change was not observed. We also performed experiments with a decane droplet and Mg^2+^ in a closed container. Even in these experiments, no remarkable and sustainable shape change and motion were observed. These results demonstrated that evaporation was required for droplet motion, and a low evaporation rate was sufficient to maintain the motion. The common pattern shown in Figure 3A is obtained probably when the evaporation rate is sufficiently low.

The oil/water interface with DEHPA and cations shows instability (spontaneous interfacial flow) when Ca^2+^, Mn^2+^, Fe^2+^, Fe^3+^, and Co^2+^ were dissolved in water (Miyaoka et al., 2012; Yasui et al., 2015). Contrastingly, this instability is not observed for Mg^2+^ and Sr^2^. This indicates that an oil droplet without instability (Mg^2+^ and Sr^2+^) can show the regular oscillations in the droplet shape. The common pattern in Figure 3A is also observed with Co^2+^, Ca^2+^, and Fe^2+^. When the spontaneous interfacial flow is generated, this common pattern is probably perturbed. This may be responsible for the less-regular pattern for Co^2+^, Ca^2+^, and Fe^2+^. The droplet with Mn^2+^ shows a regular oscillation although it causes instability. The instability is observed under restricted experimental conditions. Further studies are necessary for the elucidation of the effects of instability on droplet dynamics.

**Figure 4:**
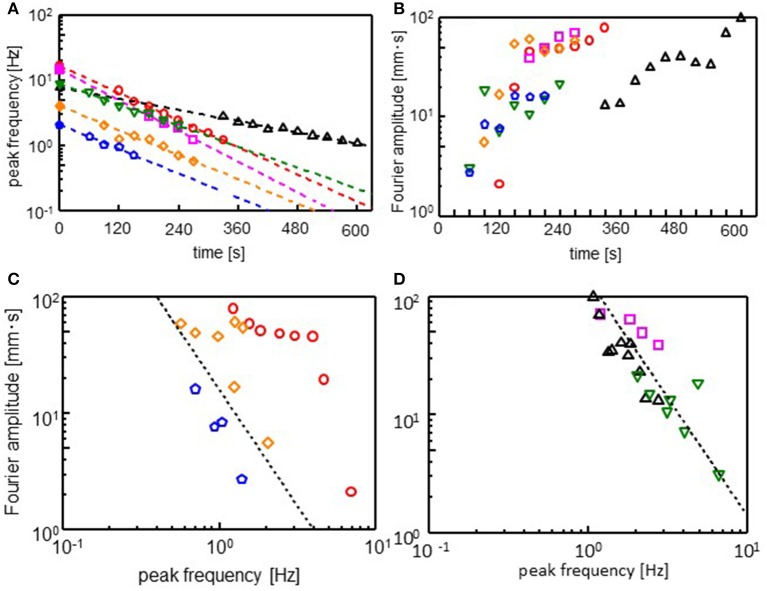
Fourier analysis of the droplet oscillations. **(A)** Peak frequency (ω_max_), **(B)** amplitude (*A*_max_) at the peak frequency, and **(C,D)** double logarithmic plot of ω_max_ and *A*_max_. Fourier transformation is performed for 30 s after the time shown in absicissa of **(A)** and **(B)**. The dotted line in **(C,D)** corresponds *A*_max_ $A_{\text{max}}\;\infty \;\omega_{\text{max}}^{-2}$. Keys are the same as those of Figure 3.

The Fourier spectra of the oscillations were calculated (see Supplementary Figure 4 for Fourier spectra). Figures 4A,B show the peak frequency (ω_max_) and its *F*ourier amplitude (*A*_max_). After an induction period, the droplet starts to oscillate. The range of ω_max_ is not large enough. However, the peak frequency appears to decay exponentially with time during the oscillation, as shown in Figure 4A. Thus, we obtain

(1) $$\begin{array}{r} \omega_{max}=\omega_{0}e^{-\frac{t}{\tau }} \end{array}$$

Here, ω_0_ is the frequency extrapolated to *t* = 0 and τ denotes the characteristic time for the frequency decay. The τ value is insensitive to the cations species, whereas it affects ω_0_. The amplitude increases to reach its maximum, as shown in Figure 4B: At the beginning of the oscillation, an oscillation with a high frequency and small amplitude governs the droplet dynamics. Subsequently, the amplitude increases, whereas the frequency decreases. This trend is observed irrespective of the cation species. The relationship between ω_max_ and *A*_max_ is shown in Figures 4C,D, which show that *A*_max_ is proportional to $\omega_{\text{max}}^{-\text{n}}$. Exponent *n* is approximately equal to 2 for all the cations. This result indicates

(2) $$\begin{array}{r} A_{max}{\omega_{max}}^{2}=p \end{array}$$

Constant *p* depends on the cation species.

**Figure 5:**
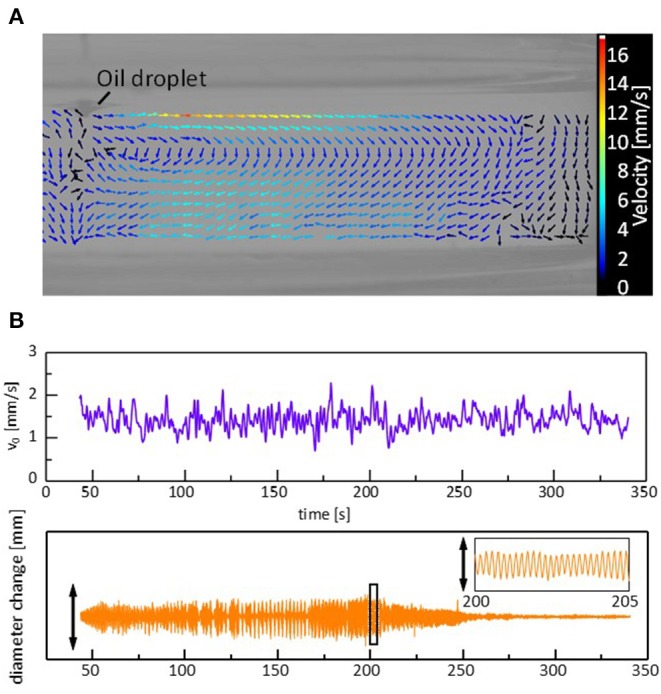
Convection caused by a sessile droplet during the regular oscillation. **(A)** Vector map of the stream lines. **(B)** Convection velocity near the droplet edge and corresponding oscillation in the droplet diameter. The vector map is at *t* = 202 s, and the speed is the average during 0.5 s around each data point. Cation is Mg^2+^.

When a droplet shows the regular oscillations, a steady convection is observed in the water phase. Supplementary Video 3, Figure 5A shows a vector map of the stream lines drawn by particle image velocimetry (PIV) analysis. The surface flow starts from the droplet edge and shows a circular pattern. The time course of a tracer speed in the vicinity of the droplet edge is shown in Figure 5B. A similar surface flow has already been pointed out (Stocker and Bush, 2003; Antoine and Pimienta, 2013), where an oil that spread over a surface was evaporated, followed by an oil supply from the droplet. In the present system, two types of explanations are possible for the origin of the flow. One is that the flow compensates for the oil evaporation from the film spread over the water surface. The other is the Marangoni effect due to the gradient in the adsorption density of DEHPA at the surface. The density may be higher near the droplet edge than that far from the droplet as the droplet contains a large amount of the surfactant, DEHPA. The droplet oscillation is caused by the force balance at the droplet edge, as discussed later. Therefore, the change in the surface (interfacial) tension should be synchronized with the droplet oscillation, as shown in the next calculation. In this case, if the Marangoni effect is the main reason for the surface flow, its velocity must be synchronized with the droplet oscillation. Considering that the velocity appears to fluctuate randomly, the surface flow is considered to be caused by the solvent evaporation from the film spread on the water surface, though the surface flow may be enhanced by the Marangoni effect.

## Model for Regular Oscillation

Here, we focus on the regular oscillation of the droplet, typically observed for Mg^2+^, Sr^2+^, and Mn^2+^. The scaling shown in Figure 3 indicates that the oscillation in case of the Ca^2+^, Co^2+^, and Fe^2+^ systems can be understood based on essentially the same mechanism.

The surface flow, as shown in Figure 5, carries the DEHPA molecules from the droplet to the water surface. Thus, the local adsorption density of DEHPA on the water surface in the vicinity of the droplet periphery, Γ(*t*), depends on time. This may be expressed by

(3) $$\begin{array}{r} \frac{d\Gamma \left(t\right)}{dt}=\frac{j{\left(v\left(t\right)-v_{0}\right)}^{2}}{\delta }-\frac{v_{0}-v\left(t\right)}{\delta }\Gamma \left(t\right) \end{array}$$

**Figure 6:**
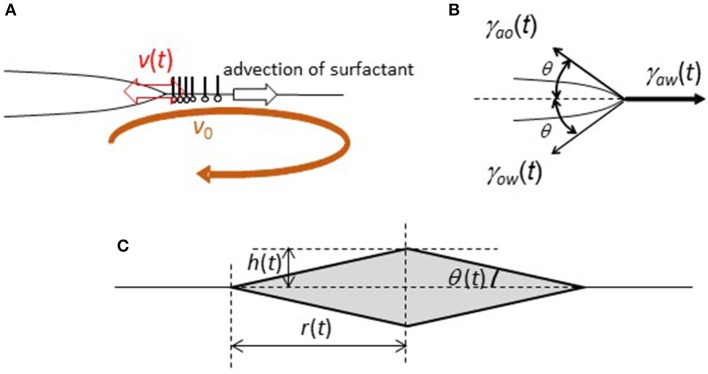
Simplified model of a sessile lens with oscillation under the surface flow. **(A)** Definition of *v(t)* and *v*_0_. **(B)** Definition of θ and the forces. **(C)** Geometry of a simplified sessile lens.

Here, *v*_0_ and *v*(*t*) denote the velocity of the surface flow and moving edge of the droplet, respectively (see Figure 6A.) We assume a constant *v*_0_ because the time change in the surface flow does not show a systematic change as shown in Figure 5B. *v*(*t*) reflects the droplet oscillation. The second term in the right hand side represents the flux of the DEHPA molecules from the droplet edge to outward. Here, a one-dimensional surface flow is considered as the simplest case. The flux of the molecules carried by the one-dimensional flow with velocity *v* is expressed by *v*Γ(*x*), where *x* denotes the one-dimensional coordinate. Then, ∂Γ(*x*)/∂*t* = –*v*∂Γ(*x*)/∂*x*. When the surface density is constant at *x*>0 and zero at *x*<0, ∂Γ(*x*)/∂*t* at *x*~0 may be approximated as –*v*(Γ−0)/δ, where Γ is the uniform density at *x*>0. δ is the characteristic length of a range, which may be regarded as the edge. Considering the droplet edge as *x* = 0, the second term can be derived, where the relative velocity of *v*_0_ against *v*(*t*) determines this flux. On the other hand, the DEHPA molecules are supplied from the droplet to the water surface near the edge. The first term corresponds to this flux. We assume that the molecules supplied from the droplet are spread over the water surface with length δ. Then, ∂Γ(*t*)/∂*t* is given by flux/δ. When the droplet edge moves with a faster or slower speed than that of the surface flow, an agitation effect enhances this mass transport. Thus, the larger the absolute value of *v*(*t*)–*v*_0_, the higher must be the flux. The mass transport coefficient enhanced by convection is usually expressed by the power of Reynolds number for the convection (Bird et al., 2006). This Reynolds number should be calculated with the relative velocity, *v*(*t*)–*v*_0_. Thus, we may consider that the mass transport coefficient is proportional to the power of the relative velocity. The exponent depends on the actual situation, and it is not easy to evaluate it in the present droplet oscillation. In the present case, however, the enhanced effect should be symmetry with respect to *v*(*t*)–*v*_0_ = 0, because the agitation effect is probably the same regardless of its sign. The simplest expression satisfying this is that the mass transport coefficient is proportional to (*v*(*t*)–*v*_0_)^2^. The *j* in Equation 3 is the proportional constant. The result of calculation is insensitive to the exponent value, if the flux is an even function of *v*(*t*)–*v*_0._ In Equation 3, the effect of diffusion on the DEHPA transport is ignored.

The droplet oscillates with the contact angle change, the schematic representation of which is shown in Figure 6B. Here, angle θ(*t*) is defined. The geometry is simplified such that the angles to the air side and water side are the same. When a droplet oscillates with angle θ(*t*), force (*F*(*t*)) that pulls the droplet edge outward is given by

(4) $$\begin{array}{r} F\left(t\right)=\gamma_{aw}\left(t\right)-\left(\gamma_{ao}+\gamma_{ow}\left(t\right)\right)\cos\theta \left(t\right) \end{array}$$

Here, γ_*ij*_(*t*) denotes the interfacial tension, which is shown in Figure 6B.

The equation of motion may be expressed by

(5) $$\begin{array}{r} \frac{d^{2}r\left(t\right)}{dt^{2}}=F\left(t\right)-\mu \left(v\left(t\right)-v_{0}\right) \end{array}$$

Here, we calculate the one-dimensional motion of a unit mass at the edge. The last term represents the viscous drag. *r*(*t*) is defined in Figure 6C. In the present experiments, Reynolds number of a droplet oscillation is approximately 10^0^-10^1^ in the order of magnitude (The density/viscosity is ~10^5^ s/m^2^ in air and 10^6^ s/m^2^ in water). The diameter of a droplet and velocity of droplet edge are ~5 mm and 5 mm/s.) Thus, the inertia term (the left hand side of Equation 5) cannot be neglected. The inertia plays an important role in for droplet oscillation in the present model, which is different from the model for contact line oscillation in camphor disk (Kitahata et al., 2008). The external force (*F*(*t*)) and dissipation term (the second term of the right hand side of Equation 5) are necessary for the oscillation caused by inertia effect.

For further calculation, one-dimensional coordinate *x* must be related to angle θ(*t*). Here, we consider a simple geometry, which is shown in Figure 6C (rhombus of revolution). This is only for simplicity. Assuming the conservation of the droplet volume, *L*^3^ = *r*(*t*)^2^*h*(*t*) is a constant. Noting that $v\left(t\right)=\frac{dr\left(t\right)}{dt}$, one obtains

(6) $$\begin{array}{r} \frac{1}{\cos\theta \left(t\right)}=\sqrt{1+{\left(\frac{L}{r\left(t\right)}\right)}^{6}} \end{array}$$

Equations 4, 5, and 6 yield

(7) $$\begin{array}{r} \frac{d^{2}r\left(t\right)}{dt^{2}}=\gamma_{aw}\left(t\right)-\frac{\gamma_{ao}\left(t\right)\;+\;\gamma_{ow}\left(t\right)}{\sqrt{1+{\left(\frac{L}{r\left(t\right)}\right)}^{6}}}-\mu \left(\frac{dr\left(t\right)}{dt}-v_{0}\right) \end{array}$$

We assume a linear dependency of γ_*aw*_(*t*) on Γ(*t*):

(8) $$\begin{array}{r} \gamma_{aw}\left(t\right)=\gamma_{aw,0}-a\Gamma \left(t\right) \end{array}$$

where γ_*aw*, 0_ denotes the air/water surface tension without DEHPA. *r*(*t*) can be calculated from Equations 3, 7, and 8 with an appropriate initial condition.

**Figure 7:**
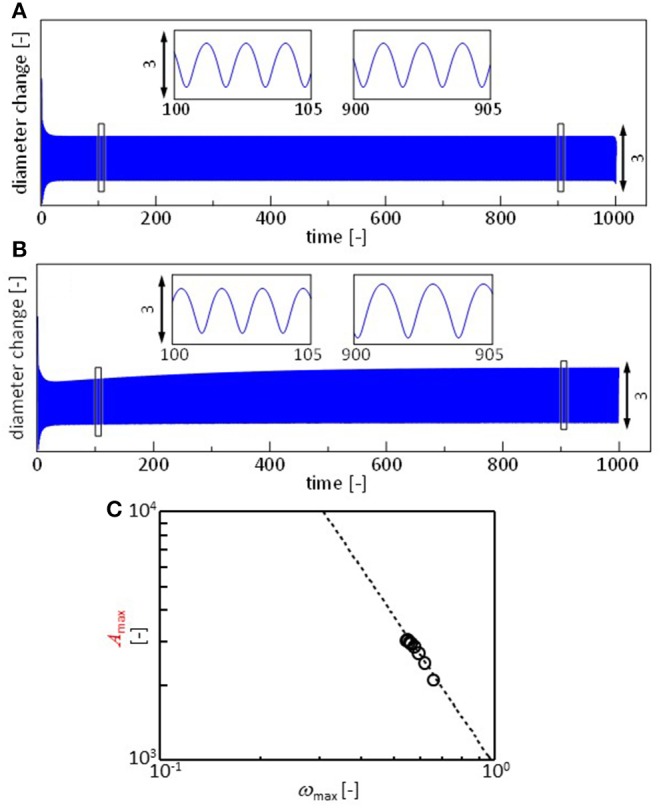
Calculation results. **(A,B)** Oscillation of droplet radius *x*. **(A)** All the parameters are constant. **(B)** The oil/water interfacial tension decays with time following Equation 9. **(C)** Fourier analysis of the result in **(B)**. This is performed similar to that for the experimental results. Fourier transformation is calculated every 30 units of the abscissa. Dashed line represents Equation 2. Paramers used for **(A)** are *a* = 1, *j* = 1, δ = 1, *v*_0_ = 2, Γ(0) = 0, *L* = 1, μ = 0.001, γ_aw, 0_ = 40, γ_ao_ = 23.5, and γ_ow, 0_ = 21. The initial value of *x* is 3. For calculation of **(B)**, γ_ow, eq_ = 18 and *k* = 0.0045. Other parameters are identical to those for **(A)**.

Figure 7A shows the *r*(*t*) calculated with Equations 3, 7, and 8, assuming that all the parameters are independent of time. The parameters for the surface and interfacial tension are adjustable, though they are determined such that their ratios become approximately equal to those in the experiments. The pattern is similar to the experimental results shown in Figure 3, in which the result of the calculation is also shown. In the experiments, the frequency and amplitude of the oscillations vary as shown in Figure 4. This may be reproduced by considering the time dependency of the parameters. The oil/water interface is free from the surfactant immediately after the droplet is placed on the surface. However, the DEHPA molecules are adsorbed at the interface, and hence, γ_ow_(*t*) must decrease in time to approach equilibrium value γ_ow, eq_. This effect may simply be expressed by

(9) $$\begin{array}{r} \gamma_{ow}\left(t\right)=\gamma_{ow,eq}+\left(\gamma_{ow,0}-\gamma_{ow,eq}\right)\times \exp\left(-kt\right) \end{array}$$

Here, γ_ow, 0_ and *k* denote the interfacial tension immediately after the droplet is placed and the rate constant, respectively. Note that the dependency of γ_aw_ on the DEHPA adsorption density is already considered with Equation 8, and γ_ao_ is practically independent of the DEHPA concentration. The exponential form of the dynamic interfacial tension has been reported in many systems (Dukhin et al., 1995). In present system, adsorbed DEHPA molecules react with cations. The products tend to aggregates at the interface. This aggregation retards the desorption. The degree of aggregation depends on cation species (Hosohama et al., 2011; Miyaoka et al., 2012). As a result of these, the interfacial tension shows the very slow dynamics (Shioi et al., 2000), which agrees with Equation 9. The result is shown in Figure 7B. The amplitude increases with time, and the frequency decreases. Fourier transformation is calculated every 30 units of the abscissa, and ω_max_ and *f* (ω_max_) are plotted in Figure 7C using Equation 2.

**Figure 8:**
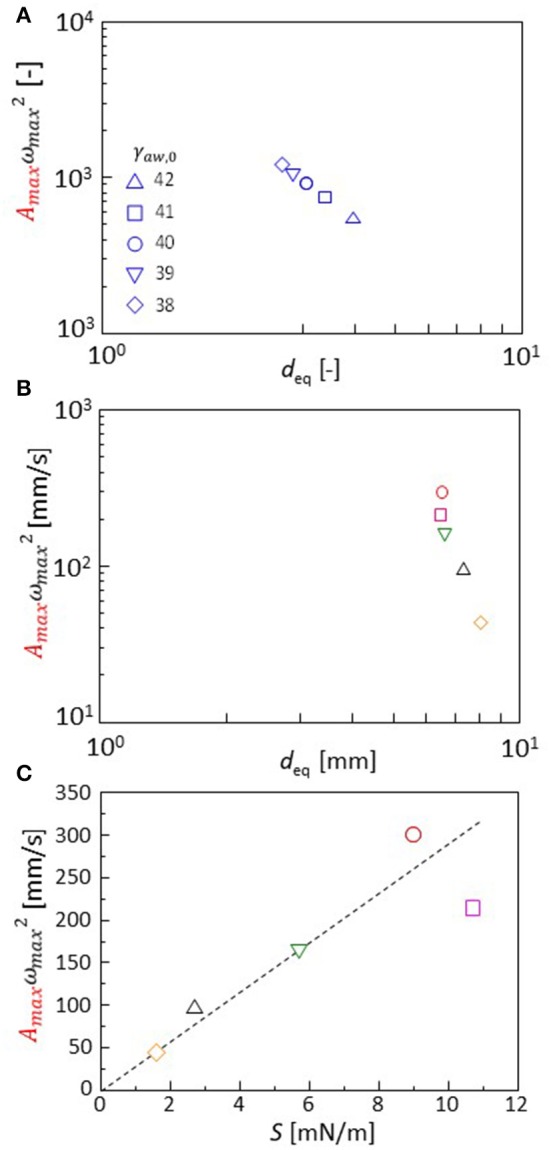
**(A,B)** *p* value *A*_maxx_$\omega_{\text{max}}^{2}$ is shown as a function of the droplet diameter. Both calculations **(A)** and experiments **(B)** are shown. **(C)** Dependency of the *p* value on extension coefficient *S* (experimental result). Keys for calculation **(A)** are obtained with variation in γ_aw, 0_ shown in the figure. The keys for experiments **(B,C)** are identical to those for Figure 3.

The Fourier transformation of the oscillation in the experiments approximately follows Equation 2. Though the range ω_max_ is narrow, this range is almost the same as that of experimental result of one type of cation shown in Figures 4C,D. The effects of the cation species on the droplet oscillation is expressed as a value of *p* using Equation 2. The cations affect the surface and interfacial tension. They must affect the droplet diameter after the oscillation has stopped. Equations 3, 7, 8, and 9 are calculated with γ_aw, 0_ (Equation 8) ranging from 38 to 42. The droplet diameter after sufficient time steps is shown as a function of the *p* value in Figure 8A. In the experiments, the droplet diameter (*d*_eq_) is measured after the droplet is dropped on the water phase in a closed container. At 300 s after the dropping, the diameter is measured. It is shown as a function of the *p* value in Figure 8B. The droplet diameter and *p* values are dimensionless in the calculation. Thus, a double logarithmic plot is used for the comparison between calculation and experiment. In both the cases, the *p* value decreases with an increase in the droplet diameter. The present model well-captures the oscillation dynamics that are observed in the experiments depending on the cation species.

Spreading coefficient *S*(= γ_aw, eq_-γ_ao, eq_-γ_ow, eq_) is a typical parameter that is associated with the droplet diameter. The *S* value is measured from the interfacial and surface tension under equilibrium of the oil and water phases. The *p* value obtained by the experiments is shown with *S* in Figure 8C. The *p* value is well-correlated with *S*. The cation species changes the spreading coefficient, which determines the characteristics in the droplet oscillation. When comparing the calculation and experiment, the droplet diameter may be used as a common parameter that may be related to the spreading coefficient: An estimation of the *S* value in calculation is not easy because the oscillation can be maintained only under a non-equilibrium state. Thus, the parameter values of the surface and interfacial tensions [γ_aw, o_, γ_ow, eq_, and γ_ao_ (time-independent)] do not correspond to the equilibrium surface and interfacial tensions observed in the experiment.

For Ca^2+^, Fe^2+^, and Co^2+^, the randomness is contained in the common pattern shown in Figure 3. In these cases, the spontaneous interfacial flow is generated at the oil/water interface (Hosohama et al., 2011). Therefore, the random fluctuation in γ_aw_(*t*) and γ_ow_(*t*) should be considered. The less-regular pattern for these cations can be explained on the basis of the present model containing the randomness.

When a flat oil/water interface is formed with DEHPA and Fe^3+^, a spontaneous interfacial flow appears intermittently. Once it is generated, it propagates over a long distance (Yasui et al., 2015). This dynamics may enhance the wetting caused by the positive spreading coefficient. Subsequently, the oil droplet is rapidly transformed into a thin film and disappears.

## Conclusion

The tuning of a droplet motion on a water surface by cations is studied. After a random motion, the droplet shows an oscillation between an expanded and a contracted state. This oscillation is driven by the evaporation of the droplet. A common pattern is shown in the oscillation. For a type of cation that does not cause instability, this common pattern is clearly observed. The frequency and amplitude in the common pattern depend on the cation species. This dependency is explained by the effect of the cation on the spreading coefficient and droplet size at equilibrium. The oscillation pattern and cation dependency are well-captured by a simple model. This study may be a basis for the chemical control of droplet dynamics on a liquid surface.

## Data Availability Statement

All datasets generated for this study are included in the article/Supplementary Material.

## Author Contributions

YM and HY found the oscillation phenomenon and performed all experiments. All calculations have been performed by YM. DY, EN-O, and AS conceived this research. YM and AS considered mathematical model. All authors discussed for this research.

### Conflict of Interest

The authors declare that the research was conducted in the absence of any commercial or financial relationships that could be construed as a potential conflict of interest.

## Supplementary Material

Supplementary Figure 1 Change in the droplet diameter.

Supplementary Figure 2 Change in droplet diameter. The drift in the droplet size over a long time is subtracted. The insets show the expanded versions of the marked squares. The scale bar is 2 mm in all the figures.

Supplementary Figure 3 Decrease in the weight of the oil. The initial volume of oil was 5 mL, which was poured in a petri-dish with diameter of 48 mm. The total weight of the dish and oil was measured every 5 min. The weight of the dish was subtracted to obtain the weight of the oil as a function of time. The petri-dish was not covered. The oil weight was scaled by its initial value.

Supplementary Figure 4 Examples of the Fourier spectra of the diameter change. Time range and cation species are shown in the graphs.

Supplementary Video 1 Droplet oscillations for six types of cations.

Supplementary Video 2 Regular oscillation of a droplet with Mg^2+^.

Supplementary Video 3 Convection in water with a droplet pinned by a pillar.
