# Supplementary material for: Ionic Tuning of Droplet Motion on Water Surface
Source: Front Chem. 2019 Nov 19;7:788. doi: 10.3389/fchem.2019.00788 (PMC6877656; doi:10.3389/fchem.2019.00788)
Supplement: Supplementary Figure 1 — Change in the droplet diameter. [file Table_1.DOCX]

**Fig. S1**

Change in the droplet diameter.

**Fig. S2**

Change in droplet diameter. The drift in the droplet size over a long time is subtracted. The insets show the expanded versions of the marked squares. The scale bar is 2 mm in all the figures.

**Fig. S3**

Decrease in the weight of the oil. The initial volume of oil was 5 mL, which was poured in a petri-dish with diameter of 48 mm. The total weight of the dish and oil was measured every 5 min. The weight of the dish was subtracted to obtain the weight of the oil as a function of time. The petri-dish was not covered. The oil weight was scaled by its initial value.

**Fig. S4**

Examples of the Fourier spectra of the diameter change. Time range and cation species are shown in the graphs.
